# The complete mitochondrial genome of *Hemigrammus bleheri* (Characiformes: Hemigrammus) and phylogenetic studies of Characiformes

**DOI:** 10.1080/23802359.2019.1681309

**Published:** 2019-11-05

**Authors:** Qi Wang, Zengliang Miao, Jian Chen, Youkun Huang, Fang Meng, Kehua Zhu, Bingjian Liu, Yifan Liu

**Affiliations:** a National Engineering Research Center for Marine Aquaculture, Zhejiang Ocean University, Zhoushan, Zhejiang, China;; b National Engineering Laboratory of Marine Germplasm Resources Exploration and Utilization, Marine Science and Technology College, Zhejiang Ocean University, Zhoushan, Zhejiang, China

**Keywords:** *Hemigrammus bleheri*, mitochondrial genome, evolutionary relationships

## Abstract

Complete mitochondrial genome of the characiform fish *Hemigrammus bleheri* was characterized in the present study. The whole mitogenome was 17,021 bp in size and consisted of 13 protein-coding genes (PCGs), 22 tRNAs, 2 rRNAs genes, a control region, and origin of light-strand replication. The proportion of coding sequences with a total length of 11,415 bp is 67.06%, which encodes 3805 amino acids. Similar to other *Hemigrammus* species, the base composition of *H. bleheri* was 29.30% for A, 25.26% for C, 16.36% for G, and 29.08% for T. All PCGs started with Met. *ND1*, *ND3*, *ND4L*, *ND6*, and *CytB* ended with TAA as the stop codon. *ND2*, *ATP8*, and *ND5* ended with TAG as a stop codon, *CO2*, *ATP6*, *CO3*, and *ND4* ended simply by T, and *CO1* ended by a single AGG. The lengths of 12S ribosomal RNA and 16S ribosomal RNA were 924 bp and 1681 bp, respectively. The length of control region (D-loop) was 1308 bp, ranging from 15,714 to 17,021 bp. The complete mitochondrial genome sequence provided here would be helpful in further understanding the evolution of characiformes and conservation genetics of *H. bleheri*.

*Hemigrammus bleheri* belongs to the family Characidae and the order Characiformes, This species is mainly distributed in the Rio Negro and Rio Meta basins. However, few reports about its basic biology data including genetic information could be indexed up to the present. In this study, we first determined the complete mitochondrial genome of *H. bleheri*, which would provide us the basic molecular data for further study on its systematics and conservation biology.

In the present study, specimens of *H. bleheri* were collected from the Rio Negro basin of Colombia (3°08′00″N, 59°54′30″W) and stored in a refrigerator at −80°C in Zhejiang Engineering Research Centre for Mariculture and Fishery Enhancement Museum (Accession number: HB180620). Total genomic DNA was extracted from muscle of three different individuals using the phenol–chloroform method (Barnett and Larson 2012; Meng et al. 2019). The calculation of base composition and phylogenetic construction was conducted by MEGA6.0 software (Tamura et al. 2013). The transfer RNA (tRNA) genes were generated with the programme tRNAs-can-SE (Lowe and Eddy 1997). The mitochondrial genome sequence of *H. bleheri* with the annotated genes was deposited in GenBank with the accession number of MK263671.

Similar to the typical mitogenome of vertebrates, the mitogenome of *H. bleheri* is a closed double-stranded circular molecule of 17,021 nucleotides including 13 protein-coding genes (PCGs), two ribosomal RNA genes, 22 tRNA genes, and 2 main noncoding regions (Boore 1999; Zhu et al. 2018). The contents of A, G, T, and C are 28.67%, 15.86%, 24.37%, and 31.10%, respectively. Most mitochondrial genes are encoded on the H-strand except for *ND6* and eight tRNA genes (*Gln*, *Ala*, *Asn*, *Cys*, *Tyr*, *Ser*, *Glu*, and *Pro*), which are encoded on the L-strand. The proportion of coding sequences with a total length of 11,415 bp is 67.42%, 13 PCGs encode 3805 amino acids in total. A-T and G-C contents of mitochondrial genome are 58.17% and 32.73% respectively.

All the PCGs use the initiation codon ATG, which is quite common in vertebrate mtDNA (Miya et al. 2001; Liu et al. 2017). *ND1*, *ND3*, *ND4L*, *ND6*, and *CytB* ended with TAA as a stop codon, *ND2*, *ATP8*, and *ND5* ended with TAG as a stop codon, *CO1* ended with a single AGG, and four incomplete termination codons (T) were found in the other four genes (*CO2*, *ATP6*, *CO3*, and *ND4*). The lengths of 12S ribosomal RNA and 16S ribosomal RNA are 924 bp and 1681 bp, which are both located in the typical positions between *tRNA-Phe* and *tRNA-Leu* (UUA), separated by *tRNA-Val* (Petrillo et al. 2006; Huang et al. 2019). The length of control region (D-loop) is 1308 bp, ranging from 15,714 to 17,021 bp.

In the neighbour-joining (NJ) tree, the result suggested that *H. bleheri* was most closely related to *Grundulus bogotensis* among all the Characiformes species included in the analysis. This result is consistent with conventional morphological taxonomy (Figure 1). 

**Figure 1.**
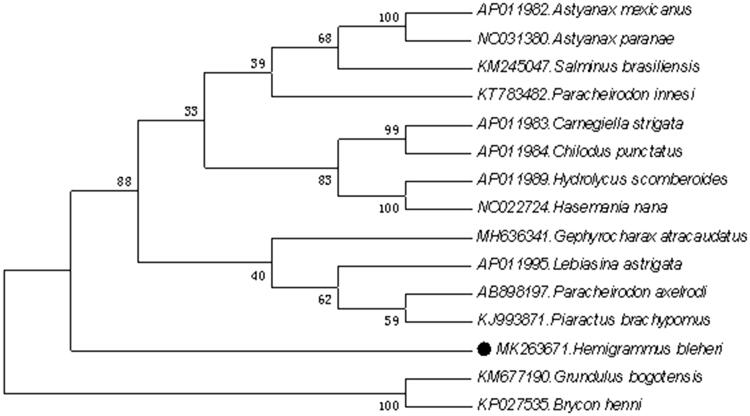
Neighbour-joining (NJ) tree of 15 Characiformes species based on 12 PCGs. The bootstrap values are based on 10,000 resamplings. The number at each node is the bootstrap probability. The number before the species name is the GenBank accession number. The genome sequence in this study is labelled with a black spot.

## References

[CIT0001] Barnett R, Larson G 2012 A phenol-chloroform protocol for extracting DNA from ancient samples. Methods Mol Biol. 840:13–19. 22237516 10.1007/978-1-61779-516-9_2

[CIT0002] Boore JL 1999 Animal mitochondrial genomes. Nucleic Acids Res. 27(8):1767–1780. 10101183 10.1093/nar/27.8.1767 PMC148383

[CIT0003] Huang Y, Liu B, Zhu K, Zhang J, Jing F, Xia L, Liu Y 2019 The complete mitochondrial genome of *Gephyrocharax atracaudatus* (Characiformes, Characidae) and phylogenetic studies of Characiformes. Mitochondrial DNA Part B. 4(1):1901–1902.

[CIT0004] Liu J, Ding Q, Gao L 2017 The complete mitochondrial genome of North Island brown kiwi (*Apteryx mantelli*). Mitochondrial DNA Part B. 2(1):1–2. 10.1080/23802359.2016.1186511 PMC7800362 33490432

[CIT0005] Lowe T, Eddy S 1997 tRNAscan-SE: a program for improved detection of transfer RNA genes in genomic sequence. Nucleic Acids Res. 10.1093/nar/25.5.955 PMC146525 9023104

[CIT0006] Meng F, Huang Y, Liu B, Zhu K, Zhang J, Jing F, Xia L, Liu Y 2019 The complete mitochondrial genome of *Lebiasina astrigata* (Characiformes: Lebiasinida) and phylogenetic studies of Characiformes. Mitochondrial DNA Part B. 4(1):579–580.

[CIT0007] Miya M, Kawaguchi A, Nishida M 2001 Mitogenomic exploration of higher teleostean phylogenies: a case study for moderate-scale evolutionary genomics with 38 newly determined complete mitochondrial DNA sequences. Mol Biol Evol. 18(11):1993–2009. 11606696 10.1093/oxfordjournals.molbev.a003741

[CIT0008] Petrillo M, Silvestro G, Nocera PPD, Boccia A, Paolella G 2006 Stem-loop structures in prokaryotic genomes. BMC Genomics. 7(1):170. 16820051 10.1186/1471-2164-7-170 PMC1590033

[CIT0009] Tamura K, Stecher G, Peterson D, Filipski A, Kumar S 2013 MEGA6: molecular evolutionary genetics analysis version 6.0. Mol Biol Evol. 30(12):2725–2729. 24132122 10.1093/molbev/mst197 PMC3840312

[CIT0010] Zhu K, Lu Z, Liu L, Gong L, Liu B 2018 The complete mitochondrial genome of *Trachidermus fasciatus* (Scorpaeniformes: Cottidae) and phylogenetic studies of Cottidae. Mitochondrial DNA Part B. 3(1):301–302. 10.1080/23802359.2018.1445480 PMC7799978 33474152

